# Adverse Immunologically Mediated Oral Mucosal Reactions to Systemic Medication: Lichenoid Tissue Reaction/Interface Dermatitis-Stomatitis, Autoimmune Vesiculobullous Disease, and IgE-Dependent and Immune Complex Reactions

**DOI:** 10.1155/2018/7645465

**Published:** 2018-06-10

**Authors:** R. A. G. Khammissa, R. Chandran, A. Masilana, J. Lemmer, L. Feller

**Affiliations:** Department of Periodontology and Oral Medicine, Sefako Makgatho Health Sciences University, Pretoria, South Africa

## Abstract

Drug-induced hypersensitivity immune reactions are exaggerated immunoinflammatory responses to allergenic components of the medications that occur in genetically susceptible subjects. The type of hypersensitivity immune response generated, whether antibody mediated or T cell mediated, or an immune complex reaction is determined by multiple factors, including the molecular characteristics of the allergen, the route of administration of the medication, the manner of presentation of the allergen by antigen-presenting cells to naïve T cells, the repertoire of the T cell receptors, and the cytokine profile within the microenvironment. This review deals with the clinical and histopathological aspects of adverse immunologically mediated oral mucosal reactions to systemic medication. We elaborate on diseases showing features of lichenoid tissue reaction/interface dermatitis-stomatitis, autoimmune vesiculobullous oral lesions, and immunoglobulin E- (IgE-) and immune complex-mediated oral reactions to drugs.

## 1. Introduction

Adverse immunologically mediated oral mucosal reactions to systemic medications are not uncommon, are variable in nature, and appear to be genetically determined. Most are mild, but some can be severe and even life threatening; so, prompt diagnosis, immediate withdrawal of the offending drug, and appropriate treatment are crucial [1].

The phenotypic diversity of drug-induced immune hypersensitivity reactions is the outcome of a complex and dynamic pathogenic process. Depending on their molecular concentration and on the context of the microenvironment, different molecular signals can mediate different or sometimes similar immunological effects; and there are interactions between multiple genes, cellular pathways, and cells. The aggregate of this integrated activity is not linear and cannot be derived from summation of the activities of the singular pathways, genes, or cells [2–4].

Susceptibility to adverse drug reactions may be increased by genetic factors determining drug metabolism, such as genetic polymorphism of cytochrome p450 enzymes, drug acetylation and methylation, and the genetic variants determining the type and magnitude of certain immune responses. These determinants include the specific human leukocyte antigen (HLA) haplotype, the T cell receptor (TCR) repertoire, or the toll-like receptor activity [1, 5]. Subjects with vascular collagen diseases, with Epstein–Barr or human immunodeficiency virus (HIV) infections, and recipients of bone marrow grafts are at increased risk of adverse drug reactions, probably because of their related immune suppression or immune dysregulation [1, 6].

Systemic medications may induce different drug-specific immunoinflammatory hypersensitivity responses including type I immunoglobulin E- (IgE-) mediated, type II IgG-mediated, type III immune complex, and type IV T cell-mediated reactions [1]. Each of these may cause a variety of oral mucosal drug eruptions [7].

In the context of drug-induced allergic reactions, the allergen may be the drug itself, a drug metabolite, a vehicle, or a preservative of the medicine. The allergen functions as a hapten, forming immunological conjugates with tissue proteins, which may then on occasion act as immunogens. In genetically predisposed subjects, allergenic medications may de novo induce immune-mediated oral mucosal diseases, may unmask latent subclinical diseases, or may aggravate the clinical course and manifestations [1, 8].

Pemphigus vulgaris, mucosal pemphigoid, linear IgA disease, lichenoid eruptions, lichen planus, lupus erythematosus, erythema multiforme, Stevens-Johnson syndrome, toxic epidermal necrolysis, and anaphylactic stomatitis are some conditions that can be induced or triggered by certain systemic medications. Therefore, in the process of diagnosing a suspected immune-mediated oral mucosal disease, the possibility of drug involvement as the aetiological factor or as a cofactor should always be considered, particularly in those cases which run an atypical clinical course [1].

Although adverse immunologically mediated oral mucosal reactions to systemic medications are generally considered to be mediated by hyperactive drug-specific T cells, it is possible that adverse drug reactions are not drug specific, but rather the result of hyperactivity of effector cells including T cells, natural killer (NK) cells, NKT cells, dendritic cells, or macrophages or of impaired immune regulatory mechanisms or both, unrelated to a specific drug. Such immune dysregulation may facilitate the development of an adverse immune reaction to a bystander drug [9]. It is also possible that reactivation of latent viruses may trigger an exaggerated virus-specific immune response that can cross-react with a bystander drug, inducing an adverse immunoinflammatory tissue reaction [10–13].

As most drug-induced immune-mediated oral diseases have clinical, histopathological, and immunological features similar to those of idiopathic immune-mediated diseases, it is to be questioned whether in both cases the outcomes are pathologically similar, or whether the drug-induced condition merely mimics the spontaneous idiopathic condition via different immunogenic mechanisms [7, 8]. In some cases, immune-mediated drug reactions resolve after withdrawal of the drug; but in other cases, despite withdrawal of the drug, the condition persists, perhaps supporting the notion of similar but differently induced immunopathogenic mechanisms [8]. The immune-mediated diseases which persist after withdrawal of the suspected causative drug should be treated as being spontaneous idiopathic immune-mediated diseases. The objectives are to relieve symptoms, to promote healing, and to prolong periods of remission [14]. In general, highly potent topical or systemic glucocorticosteroids are the main pharmacological agents of choice, but severe cases of immune-mediated oral diseases may necessitate the use of other agents with immunosuppressive and/or anti-inflammatory properties [15].

When evaluating a patient with a putatively immune-mediated oral mucosal disease who is also taking systemic medications, the question is whether the condition is idiopathic or drug related. To complicate matters, older subjects are often taking several drugs, each of which may be inducing an immune reaction, which may be affected by drug interaction, and changes in the drug regimen create uncertainty as to whether currently or previously used drugs may be implicated. Therefore, reaching a conclusion about whether an immune-mediated reaction is drug-induced or is idiopathic can be difficult if not impossible [12, 13, 16, 17]. Yet another complication is the possibility of exposure to industrial, occupational, or even household agents that can induce or trigger immune responses, thus influencing the pathogenesis and course of immune-mediated oral conditions [18].

Tissue reaction to systemic medications may or may not be predictable (Table 1) [19], but this review deals only with the clinical aspects and the pathogenesis of immunologically mediated adverse oral mucosal reactions to systemic medications.

## 2. Allergic Sensitization to Drugs

Only a small proportion of subjects who are genetically susceptible will develop adverse drug-induced, immunologically mediated oral mucosal reactions to systemic medications. Sensitization to an allergenic drug is necessary for the generation of T cell-mediated and antibody-mediated allergic immune reactions. The sensitization cascade starts with the detection and processing of the allergen by antigen-presenting cells of the myeloid lineage and is then followed by the presentation of the allergen to naïve T cells in the regional lymph nodes in the context of major histocompatibility complex (MHC) molecules. Depending on the molecular characteristics of the allergen, the route of administration of the medication, the repertoire of the T cell receptors, and the cytokine profile in the microenvironment, the naïve T cells will differentiate into distinct effector T cell subsets, either T helper 1 (Th1), Th2, Th17, or regulatory T cells with their distinct associated cytokine profiles [20, 21]. However, polarized T cell populations maintain functional plasticity with the capacity to produce some cytokines that are not considered lineage specific [22]. Sometimes the drug-peptide complex may directly activate T cells by interacting with their receptors without prior priming in lymph nodes [1].

In the context of allergic reactions, Th1 polarization with priming of antigen-specific CD4+ T cells and CD8+ cells occurs in the background of Th1 cytokines including interleukin-2 (IL-2) and interferon-*γ* (IFN-*γ*), leading to recruitment and activation of eosinophils and monocytes/macrophages that together with the primed T lymphocytes, generate a T cell-mediated delayed immune hypersensitivity reaction [1]. On the other hand, Th2 polarization with the synthesis of allergen-specific IgE by B lymphocytes on the background of Th2 cytokines such as IL-4 and IL-5 leads to the recruitment and sensitization of effector cells including basophils, eosinophils, and mast cells, generating an IgE-mediated immediate immune hypersensitivity response [20].

The various drug-induced, immunologically mediated oral mucosal conditions are phenotypically distinct although their immunopathogenic mechanisms may be the same and are heterogeneous in their genetic determinants, and their aetiopathogenesis is influenced by gene-gene interactions and environmental factors such as infective agents [6, 10]. However, in the case of oral lichenoid tissue reactions such as allergic contact stomatitis, in oral lupus erythematosus or in classical lichen planus, the clinical pictures may be similar despite different pathogenic mechanisms. The same applies to drug-triggered oral autoimmune disorders including the pemphigus and pemphigoid group of diseases or linear IgA disease. The same drug, in the background of different genetic susceptibility or environmental factors may trigger different immune mechanisms, each with its own distinct cytokine profile, resulting in the great diversity of the clinical manifestations [1].

## 3. Lichenoid Tissue Reactions/Interface Dermatitis-Stomatitis

A number of clinically diverse immunopathogenic mucocutaneous inflammatory disorders including lichen planus, allergic lichenoid reactions, lichenoid graft versus host disease, lupus erythematosus, fixed drug eruptions, Stevens-Johnson syndrome (SJS), and toxic epidermal necrolysis (TEN), whether idiopathic or induced or triggered by systemic medications, have similar histopathological features. These histopathological features include necrosis, apoptosis, and disorganisation of basal epithelial keratinocytes with cellular changes described as liquefactive/vacuolar associated with a band-like inflammatory cell infiltrate at the dermal/epidermal interface [23, 24] comprising mononuclear inflammatory cells, predominantly activated T lymphocytes but also macrophages and dendritic cells, and less common neutrophils, eosinophils, and natural killer cells. The infiltrating lymphocytes are in many cases so numerous as to obscure the epithelial-connective tissue junction and are thought to directly cause the epithelial damage [23, 25]. All mucocutaneous diseases with the clinical features of lichenoid tissue reactions and the histopathological features described above are termed “lichenoid tissue reaction/interface dermatitis,” and to the opinion of the authors, “stomatitis” can be appended [23, 24].

It appears that type 1 IFN-*α*/*β* secreted by plasmacytoid dendritic cells within the inflammatory infiltrate of oral lichen planus and cutaneous lupus erythematosus plays an important role in the pathogenesis of these particular diseases. Type 1 IFN mediates Th1-directed immunoinflammatory reactions and recruitment of cytotoxic T cells to the inflamed tissue and upregulate the expression of cytotoxic agents by cytotoxic T cells and NK cells. This amplifies the immunoinflammatory reactions of oral lichen planus and cutaneous lupus erythematosus [26–28].

The degeneration of the basal cell layer of the epithelium in lichenoid tissue reaction/interface dermatitis-stomatitis is a consequence of necrosis of basal keratinocytes characterized by rapid cytoplasmic swelling with breakdown of intracellular organelles and rupture of the cell membrane and/or of apoptosis of basal keratinocytes characterized by chromatin condensation at the nuclear membrane, compaction of intracellular organelles, and cell shrinkage forming apoptotic bodies. This process of cell death directly caused by products of infiltrating lymphocytes blurs the line between the concept of apoptosis and necrosis [23–25, 29, 30].

It has been established that both antigen-specific CD4+ and CD8+ T cells, cytokines and chemokines secreted by local keratinocytes, and dedicated antigen-presenting cells mediate the death of keratinocytes and the related tissue damage. The specific roles that CD4+ T cells and CD8+ T cells play in the pathogenesis of diseases of the lichenoid tissue reaction/interface dermatitis-stomatitis diseases are not well defined, but it is clear that both are essential for the generation of the immune reactions which cause their diverse clinical phenotypes. It appears that lymphocyte-initiated apoptosis of keratinocytes plays an essential role in the pathogenesis of lichenoid tissue reaction/interface dermatitis-stomatitis diseases. In this context, apoptosis may be induced by direct interaction of the cell death receptor Fas/CD95 expressed by keratinocytes and Fas-ligand (Fas L) expressed by effector cells such as T cells and NK cells or indirectly by secretory pathways including release into the microenvironment of tumour necrosis factor-*α* (TNF-*α*), soluble Fas L, or cytolytic/cytotoxic granules containing the pore-forming perforin proteins, granulysin, and granzymes which are all members of a family of serine proteases [31–34]. Intracellularly, caspases, a family of cysteine proteinases, ultimately drive the process of apoptosis [30, 34, 35].

Fas-FasL interactions mediated by both antigen-specific CD4+ and CD8+ T cells induce apoptosis of keratinocytes and cytokines secreted by CD4+ T cells including IFN-*γ* and TNF-*α* contribute to the tissue damage. It appears that cytotoxic CD8+ T cells containing perforin, granzyme, and granulysin are the principal effectors of death of basal keratinocytes either by apoptosis or by necrosis. Perforin makes pores in the cell membrane of target cells, paving the way for granzyme B containing endosome-like vesicles to enter the target cells, and subsequent granzyme release degrades DNA molecules and induces apoptosis. NK cell-mediated cytotoxicity and NKT cell responses also contribute to tissue damage in some of the lichenoid tissue reaction/interface dermatitis-stomatitis diseases [24, 25, 30, 33, 35–37].

Th17 cells with their associated IL-17 and IL-22 cytokines may play a role in the pathogenesis of some lichenoid tissue reaction/interface dermatitis-stomatitis diseases, and CD4+ T regulatory cells are important in regulating the CD4+ and the CD8+ T cell immunoinflammatory responses [24].

Antigen-bearing keratinocytes in the basal/parabasal cell layers of the epithelium are thought to be the target of the immune reaction in lichenoid tissue reaction interface dermatitis-stomatitis but the nature of the target molecule is not known. This might be a self-antigen such as Ro/SSA or La/SSB as in lupus erythematosus or a bioactive drug molecule which serves as a hapten to form immunogenic conjugates with self-proteins or even an environmental element such as an infective agent mimicking the molecular structure of a self-antigen and generating a cross-reactive immune response [23, 24]. Regardless of the precise immune mechanism involved, it is clear that imbalances in the immune responses caused either by hyperactive effector immunocytes or by reduced functional activity or number of regulatory T cells, or both, result in the development of adverse drug reaction [9]. Indeed, it has been demonstrated that in lupus erythematosus and in SJS/TEN, the immune regulatory mechanisms are impaired, and consequently, the function of hyperactive T cells is uncontrolled; so, a severe immunoinflammatory reaction to medication may develop [9, 28].

### 3.1. Fixed Drug Reactions

Following a primary episode of allergic skin/mucosal eruption induced by a drug-specific T cell-mediated hypersensitivity immune reaction in response to exposure to a systemic drug, reexposure to the same or to a chemically closely related drug may induce a recurrent eruption at the same site. This “fixed drug eruption” is probably because CD8+ memory T cells persist at the site of the initial eruption triggering further eruptions on subsequent exposures [38]. These memory cells involved in the pathogenesis of fixed drug eruptions may well be resident lymphocytes involved in controlling latent human herpes virus infection [23, 38]. A fixed drug-induced reaction of the oral mucosa is thus an immunoinflammatory condition clinically manifesting as zones of erythema and oedema, which may progress to erosions or vesicles. The labial mucosa is the oral site most frequently affected [7].

### 3.2. Lichen Planus/Lichenoid Reaction

Oral lichen planus is an idiopathic immunoinflammatory mucocutaneous condition, but sometimes, in genetically susceptible subjects, it may be triggered by certain systemic medications in which case it is termed lichenoid drug eruption or drug-induced lichen planus. Before a lichenoid drug eruption occurs, there is usually an unpredictable delay varying from a few days to several years of use of a particular drug and once it has developed, the disease may persist long after the drug is discontinued. Idiopathic oral lichen planus and drug-induced oral lichen planus/lichenoid reaction are clinically and histopathologically similar, and lichenoid drug eruptions which mimic lichen planus may even appear bilaterally further increasing the resemblance to lichen planus [16, 17, 39, 40].

Lichenoid drug eruptions are usually ill-defined erythematous erosive lesions with a lichen-like hyperkeratosis, and in both lichen planus and lichenoid tissue reaction, the cytotoxic CD8+ T cells outnumber the CD4+ T helper cells on the background of a Th1 cytokine milieu [23]. Direct immunofluorescence studies show deposits of fibrinogen, fibrin, C3, and sometimes IgM at the basement membrane zone [41].

It is possible that in drug-induced lichen planus/lichenoid reaction, CD8+ T cells may be activated by a drug molecule expressed by basal keratinocytes in association with MHC class I molecules and that the activated cytotoxic CD8+ T cells may trigger necrosis or apoptosis of keratinocytes via the Fas-Fas L, granulysin, or perforin/granzyme pathways [23, 42, 43]. One cannot discard the idea that in what appears to be a drug-induced lichenoid reaction, the drug may in fact have triggered an immunoinflammatory response that then unmasks preexisting subclinical oral lichen planus [44].

### 3.3. Lupus Erythematosus

Cutaneous lupus erythematosus is a chronic mucocutaneous immune-mediated inflammatory disorder brought about by complex interactions between intrinsic factors including susceptible genes determining immune responses and clearance of apoptotic cells on the one hand and extrinsic factors including ultraviolet radiation, infectious agents, and certain drugs on the other hand. Increased apoptosis, impaired clearance of apoptotic cells, impaired immune regulatory functions with consequent lower activation thresholds of T and B lymphocytes, reactive antibodies against intracellular constituents of nucleosomal DNA proteins and ribonucleoproteins, and dysregulation of the cytokine network, particularly the overexpression of TNF-*α* and type 1 interferons, all together drive the inflammatory process causing the tissue damage in lupus erythematosus [27, 28, 45].

Upregulation of expression of CD1d receptor by keratinocytes and by antigen presenting cells secondary to epithelial injury, or to inflammation at the basement membrane zone, may cause inactivation of invariant natural killer T (iNKT) cells by CD1d-bound glycolipid antigens. iNKT cells are a functionally versatile subset of T lymphocytes which by secreting into the local microenvironment, a variety of soluble biological factors, and by directly interacting with various adaptive or innate cells, has the capacity either to enhance or to regulate immunoinflammatory processes [46–48]. Furthermore, iNKT cells which also express perforin, granzyme B, and Fas L are believed to play a role in the pathogenesis of cutaneous lupus erythematosus [46, 47].

Lesions of oral lupus erythematosus manifest as well-defined atrophic, erosive, or ulcerated areas with radiating keratotic striae and surrounding telangiectasia, predominantly affecting the buccal mucosa, but also the gingiva, labial mucosa, and the vermilion border of the lip. Although discoid lupus erythematosus may affect only the oral mucosa, it usually occurs together with skin lesions [27].

The mechanisms by which drugs may trigger lupus erythematosus are not well understood, but it has been suggested that certain drugs induce the production of the cytokines TNF-*α* and IFN-*α* which have the potential to stimulate and to increase the autoreactive capacity of B and T lymphocytes, or alternatively, some drugs by promoting epigenetic modifications can dysregulate T lymphocyte gene expression, resulting in the T cells becoming autoreactive [27, 28].

### 3.4. Erythema Multiforme

Erythema multiforme is an acute immune-mediated mucocutaneous blistering disease. The oral mucosa is involved in up to 70% of cases and not infrequently may be the only site affected. Periods of disease activity usually last 10–20 days; remissions last months to several years. On average, within a period of 10 years, there are six episodes of disease activity [49].

Oral erythema multiforme has a predilection for the lips and the anterior part of the mouth but can affect any part of the nonkeratinized oral mucosa. The primary erythematous macules rapidly become blisters and soon rupture leaving painful diffuse multifocal erosions or superficial ulcers surrounded by zones of erythema. The lips are invariably hyperaemic, eroded or ulcerated, split, bleeding, and crusted. There is interference with eating, swallowing, and speech [49–52].

Erythema multiforme may be idiopathic, but most cases are associated with herpes simplex virus or *Mycoplasma pneumoniae* infections or with any one of a variety of drugs [52]. In susceptible subjects, drug-induced oral erythema multiforme usually occurs within a few days of starting a systemic medication and resolves upon its discontinuation [53]. It is likely that a cytotoxic CD8+ T cell immune reaction to a drug metabolite within the oral epithelium plays a fundamental role in the initiation of drug-induced oral erythema multiforme [50].

### 3.5. Stevens-Johnson Syndrome (SJS) and Toxic Epidermal Necrolysis (TEN)

The current literature distinguishes between SJS and TEN but nevertheless draws attention to the similarities and to their often-overlapping presentation. SJS and TEN are related life-threatening immune-mediated conditions characterized by epithelial blisters, which progress to severe, diffuse mucocutaneous erosions with diffuse detachments of necrotic epithelium accompanied by fever and sometimes by toxic visceral effects. In SJS, there is less than 10% of the skin detachment and in TEN, more than 30%, and when there is between 10% and 30% of skin detachment, the condition is conveniently termed “SJS-TEN overlapping syndrome” [19, 54]. All cases of TEN and about 75% of SJS are induced or triggered by systemic medications within one to eight weeks of introducing the offending drug [19].

Drug-specific cytotoxic T cells reactive to keratinocytes, NK cells, the cytokines TNF-*α* and IFN-*γ*, and increased levels of perforin, granzyme B, and granulysin are all found in the lesional blisters of SJS/TEN. This suggests that the necrosis of keratinocytes is mediated predominantly by drug-specific HLA-restricted cytotoxic T lymphocytes via a perforin/granzyme-mediated pathway [1, 19, 31, 33, 52, 55].

In addition to necrosis of keratinocytes, the tissue damage in SJS/TEN is also characterized by widespread keratinocyte apoptosis mediated by interactions between the death receptor Fas and its ligand Fas L and by the cytotoxic protein granulysin secreted by activated T cells and NK cells [19, 32, 33, 52, 54, 55].

## 4. Drug-Induced IgE-Dependent Immune Hypersensitivity Response

IgE-dependent anaphylactic reactions are caused by biological mediators of inflammation released either by tissue mast cells or by circulating basophils or by both. Mast cells and basophils express the high-affinity Fc receptor for IgE, the FceRI. Binding of allergen-specific IgE to FceRI results in sensitization of the cells enabling effector responses so that subsequent exposure to the specific allergen may result in its crosslinking to IgE molecules bound to FceRI-bearing cells. This interaction results in the almost immediate release of preformed biological mediators such as histamine, leukotrienes, and prostaglandins which drive the IgE-dependent hypersensitive effector reactions [20, 56, 57].

In response to stimulation by allergen-specific IgE, mast cells may also de novo produce and release a variety of cytokines, chemokines, and growth factors which have the capacity to recruit and activate innate immune cells including eosinophils, neutrophils, and basophils. The functional activity of mast cells varies at different anatomical sites and is dictated by the cytokine profile and the cells in the specific local microenvironment [58].

Certain drugs have the capacity to directly trigger degranulation of mast cells/basophils thus bringing about pseudoallergic/anaphylactoid reactions by nonimmunological activation of effector pathways [1]. These pseudoallergic anaphylactoid reactions can occur on first exposure to the drug, developing within minutes [1, 19].

### 4.1. Drug-Induced Anaphylactic Reactions of the Oral Mucosa and Surrounding Tissues

Angioedema and anaphylactic stomatitis are manifestations of drug-induced, IgE-mediated allergic reactions [7]. Angioedema is a localized cutaneous/mucosal oedematous swelling brought about by a temporary increase in vascular permeability mediated by vasoactive biological agents. Histopathologically, there is a perivascular infiltrate of eosinophils and lynphocytes with, and lymphocytes with an increase in the endothelial intercellular spaces and separation of perivascular collagen bundles. Drug-induced histamine release from mast cells/basophils occurs as a result either of an IgE-dependent allergic reaction or of direct IgE-independent degranulation of these cells resulting in histaminergic angioedema. The face is most commonly affected, and occasionally also the pharynx, larynx, oropharynx, or the oral tissues. Angioedema of the mucosa of the upper aerodigestive tract may be life threatening because of the risk of upper airway obstruction [59].

## 5. Immune Complex-Mediated Hypersensitivity Reaction

Antigen-antibody complexes cause tissue damage by eliciting inflammation at the sites of their deposition. The pathogenesis of this inflammation occurs in three phases: firstly, the formation of antibodies against either endogenous- or exogenous-free circulating antigens and formation of antigen-antibody complexes; secondly, deposition of the complexes in vessel walls with fixation of complement which together with Fc receptors initiates leukocyte recruitment and activation; and thirdly, acute inflammation with tissue damage. The principal morphological manifestation of immune complex disease is acute necrotizing vasculitis with fibrinoid necrosis of the vessel wall and an intense infiltrate of neutrophils extending through the vessel wall into the perivascular zone, where they degenerate into nuclear dust. In the skin, this is referred to as leukocytoclastic vasculitis [60].

Although immune complex disease can be systemic, involving many organs, in the context of this review, we deal with localized deposition of immune complexes in cutaneous and mucosal vasculature. Immunoglobulins and complement components can only be demonstrated in early lesions; but in up to 40% of cases, the causative immune complex cannot be identified. In most cases, the lesions are induced either by drugs or by infective agents [1]. In the classic form of drug-induced immune complex reaction, there is deposition of immune complexes with the activation of complement, resulting in fever, arthritis, oedema, or skin and mucosal lesions. Typically, complement-mediated immune complex tissue damage develops some six days after exposure to the allergen, this being a period required for the production of the drug-specific antibodies [1]. Drug-based immune complexes may activate plasmacytoid dendritic cells expressing Fc receptors which then secrete INF-*α*, contributing to the adverse immunoinflammatory drug reaction [61].

In the oral mucosa, minor and major aphthous ulcers, aphthous-like Behcet's disease, and erythema multiforme are said to be examples of immune complex hypersensitivity, but while perivascular lymphocytic cuffing and neutrophilic infiltration of the epithelium have been sometimes described, true vasculitis and immune complexes are hardly ever seen [60, 62].

## 6. Autoimmune Vesiculobullous Diseases

Pemphigus and pemphigoid groups of diseases and linear IgA disease are autoimmune mucocutaneous vesiculobullous conditions in which the oral mucosa is not infrequently affected. These diseases may either be idiopathic or be triggered by a variety of extrinsic agents such as viruses or medications and are clinically characterized by inflammation, blisters, or erosions [7, 10].

It is possible that genetically determined potential autoimmune mucocutaneous vesiculobullous diseases that have been suppressed by intrinsic factors can be triggered by drugs that destabilise the immune network in such a way that the subclinical disease becomes overt [10]. Some allergenic drugs may bond to epithelial or basement membrane zone proteins either to change their antigenicity or to form a hapten-peptide complex, eliciting in a genetically predisposed subject, an immune hypersensitivity reaction, bringing about blisters of the oral mucosa [18, 63, 64]. Another possibility is that drug-induced immunoinflammatory reactions cause local immune dysregulation which in turn leads to loss of control of viral replication and reactivation of latent viruses. In response to the subclinical viral infection, there is upregulation of cytokine expression promoting the development of autoimmune mucocutaneous blistering diseases owing to loss of immune tolerance with increased autoreactive capacity of immune cells to self-antigens [10].

Certain drugs, by nonimmunological mechanisms, can cause epithelial damage by dysregulating the activity of enzymes essential for the function of keratinocytes and for keratinocyte adhesion to one another and to the basal lamina. Drugs containing thiol groups have the biochemical capacity to cause intraepithelial and epithelial-laminal propia separation with the development of oral mucosal blisters [12, 18, 63, 65]. Furthermore, in susceptible subjects, some drugs can stimulate keratinocytes to produce and release cytokines such as TNF-*α* and IL-1*α* which can also result in acantholysis [12].

It has been suggested that in the category of drug-induced immune-mediated mucocutaneous diseases, there are two possible responses: one is self-limiting and develops some time after starting the medication and resolving when the drug is withdrawn and the other is triggered rather than induced by a drug and runs a persistent clinical course despite the withdrawal of the offending drug [64, 65].

The clinical, histopathological, and immunological features of idiopathic and drug-induced autoimmune-mediated mucocutaneous blistering diseases are indistinguishable [63, 66]. However, it appears that drug-induced mucosal blistering diseases usually affect younger subjects, undergo remission after withdrawing the offending drug, and respond more favourably to treatment than do idiopathic blistering diseases [63, 64]. Furthermore, it has been reported that subjects with drug-related mucocutaneous blistering diseases less frequently show circulating autoantibodies to self-antigens than do subjects with idiopathic mucocutaneous blistering diseases [18].

### 6.1. Pemphigus Vulgaris

Pemphigus vulgaris is a potentially fatal autoimmune mucocutaneous blistering disease. The oral mucosa is commonly involved and occasionally is the first site of presentation. The disease runs a prolonged course, with the blisters rupturing soon after formation leaving ill-defined, irregularly shaped, painful superficial erosions or ulcers with marginal tags of epithelium that can be peeled off beyond the margins of the lesion. The lesions are slow to heal, interfering with eating, drinking, swallowing, and speech, with continual formation of new blisters [67, 68].

In pemphigus vulgaris, there is binding of autoreactive IgG to desmosomal proteins (desmogleins) causing the loss of keratinocyte cohesion. This results in acantholysis with the formation of suprabasal intraepithelial blisters with free-floating epithelial cells within the blister (Tzanck cells). The basal keratinocytes constitute the floor of the blister and do not lose their attachment to the basement membrane zone. Neutrophils and eosinophils may be present in the superficial portion of the lamina propria [67].

It has been reported that up to 50% of first-degree relatives of subjects with overt pemphigus have subclinical immune reactions characteristic of pemphigus, including circulating autoantibodies to desmosomal proteins and epithelium-bound autoantibodies, without having any overt signs of disease [65]. Genetic susceptibility, viral infection, and certain medications are some factors that play roles in the pathogenesis of pemphigus vulgaris and that have the capacity to influence its clinical manifestations and course. It is probable that in subjects with subclinical characteristics of pemphigus, there are some protective genetic factors that prevent the development of the overt disease [65].

### 6.2. Mucosal Pemphigoid

Mucosal pemphigoid is an autoimmune blistering disease, which often starts in the mouth, and is sometimes limited to the oral mucosa. It is characterized by tissue-bound autoreactive antibodies against structural proteins of the basement membrane zone and of hemidesmosomes that mediate the recruitment of inflammatory and immune cells with the generation of chronic inflammation. This results in the detachment of basal keratinocytes from the underlying basement membrane zone with the formation of subepithelial blisters [69, 70]. Rupture of the blisters leaves painful, irregular, wide-spread superficial ulcers, and the erythematous denuded tissue is covered with a fibrinous pseudomembrane. The buccal mucosa, palate, and the gingiva are the most frequently affected oral sites. The lesions heal without scarring [69].

Characteristic microscopic features of mucosal pemphigoid include a subepithelial chronic inflammatory cell infiltrate of eosinophils, lymphocytes, and neutrophils and a linear deposition of immunoglobulins and complement within the basement membrane zone [69].

Usually, mucosal pemphigoid is idiopathic. However, the configuration of hemidesmosomal and certain basement membrane zone proteins may be altered by drugs or viruses resulting in the development of compound autoimmunogens generating autoimmune reactions. In genetically susceptible subjects, drugs or viruses may also modify preexisting subclinical immune reactions, thus triggering or promoting the development of the disease.

### 6.3. Linear IgA Disease

Linear IgA disease is an uncommon autoimmune mucocutaneous disorder defined by a linear pattern of IgA deposited in the basement membrane zone. The disease may be idiopathic or drug-induced but the clinical, histopathological, and immunological features are similar in both forms. Oral lesions are not uncommon, manifesting as erythematous macules, subepithelial blisters, or erosions with pain or a burning sensation [71–73].

Histopathological examination shows vacuolar degeneration of basal cell keratinocytes with subepithelial blistering and a predominantly T cell- and neutrophil-mixed cell infiltrate in the superficial portion of the lamina propria. The self-antigens targeted in linear IgA disease are proteins in the lamina lucida and lamina densa of the basement membrane zone, and the tissue damage is caused by T lymphocytes and neutrophils and by cytokines and inflammatory mediators released into the microenvironment. In patients with the idiopathic form of the disease, circulating antigen-specific antibodies may or may not be present but are absent in the drug-induced form [71–73].

In drug-induced linear IgA disease, signs and symptoms usually develop within the first month of using the drug and resolve spontaneously some time after discontinuation of the drug [73].

## 7. Conclusion

Adverse immunologically mediated oral mucosal reactions to systemic medications are exaggerated immunoinflammatory responses to allergenic components of the medication occurring in genetically susceptible persons. Lichenoid tissue reaction/interface stomatitis diseases may be idiopathic or induced by systemic medications. Antigen-bearing keratinocytes in the basal/parabasal cell layers of the oral epithelium are thought to be the target of the immune reaction in these diseases, but the nature of the target molecules is not clear.

IgE-dependent and immune complex reactions and vesiculobullous diseases are immunopathogenic reactions usually developing when the medication has been used for some time and resolves when it is withdrawn or may be triggered rather than being induced by medication, in which case the disease runs a persistent clinical course despite the withdrawal of the offending drug.

## Figures and Tables

**Table 1:** Predictable and unpredictable reactions to systemic medications: general considerations [19].

Predictable drug effects	Unpredictable drug reactions
(i) Expected pharmacological action	(i) Drug intolerance^X^
(ii) Unavoidable side effects	(ii) Idiosyncracy^Y^
(iii) Drug toxicity	(iii) Immunologically mediated
(iv) Adverse effects of known drug-drug interactions	

^X^Known drug reactions occurring at much lower doses or blood concentration. ^Y^Unexpected drug reactions which do not occur in the vast majority of subjects taking this particular medication.
